# External Validation of the Prostate Biopsy Collaborative Group Risk Calculator and the Rotterdam Prostate Cancer Risk Calculator in a Swedish Population-based Screening Cohort

**DOI:** 10.1016/j.euros.2022.04.010

**Published:** 2022-05-19

**Authors:** Jan Chandra Engel, Thorgerdur Palsdottir, Donna Ankerst, Sebastiaan Remmers, Ashkan Mortezavi, Venkatesh Chellappa, Lars Egevad, Henrik Grönberg, Martin Eklund, Tobias Nordström

**Affiliations:** a Department of Clinical Sciences at Danderyd Hospital, Karolinska Institutet, Stockholm, Sweden; b Department of Medical Epidemiology and Biostatistics, Karolinska Institutet, Stockholm, Sweden; c Department of Mathematics and Life Science Systems, Technical University of Munich, Munich, Germany; d Department of Urology, Erasmus MC Cancer Institute, Erasmus University Medical Center, Rotterdam, The Netherlands; e Department of Urology, University Hospital Basel, Basel, Switzerland; f Department of Oncology-Pathology, Karolinska Institutet, Stockholm, Sweden

**Keywords:** Prostate cancer, Risk calculators, External validation study, Recalibration of risk prediction tools, Prostate cancer screening

## Abstract

**Background:**

External validation of risk calculators (RCs) is necessary to determine their clinical applicability beyond the setting in which these were developed.

**Objective:**

To assess the performance of the Rotterdam Prostate Cancer RC (RPCRC) and the Prostate Biopsy Collaborative Group RC (PBCG-RC).

**Design, setting, and participants:**

We used data from the prospective, population-based STHLM3 screening study, performed in 2012–2015. Participants with prostate-specific antigen ≥3 ng/ml who underwent systematic prostate biopsies were included.

**Outcome measurements and statistical analysis:**

Probabilities for clinically significant prostate cancer (csPCa), defined as International Society of Urological Pathology grade ≥2, were calculated for each participant. External validity was assessed by calibration, discrimination, and clinical usefulness for both original and recalibrated models.

**Results and limitations:**

Out of 5841 men, 1054 (18%) had csPCa. Distribution of risk predictions differed between RCs; median risks for csPCa using the RPCRC and PBCG-RC were 3.3% (interquartile range [IQR] 2.1–7.1%) and 20% (IQR 15–28%), respectively. The correlation between RC risk estimates on individual level was moderate (Spearman’s *r* = 0.55). Using the RPCRC’s recommended risk threshold of ≥4% for finding csPCa, 36% of participants would get concordant biopsy recommendations. At 10% risk cut-off, RCs agreed in 23% of cases. Both RCs showed good discrimination, with areas under the curves for the RPCRC of 0.74 (95% confidence interval [CI] 0.72–0.76) and the PBCG-RC of 0.70 (95% CI 0.68–0.72). Calibration was adequate using the PBCG-RC (calibration slope: 1.13 [95% CI 1.03–1.23]), but the RPCRC underestimated the risk of csPCa (calibration slope: 0.73 [0.68–0.79]). The PBCG-RC showed a net benefit in a decision curve analysis, whereas the RPCRC showed no net benefit at clinically relevant risk threshold levels. Recalibration improved clinical benefit, and differences between RCs decreased.

**Conclusions:**

Assessment of calibration is essential to ensure the clinical value of risk prediction tools. The PBCG-RC provided clinical benefit in its current version online. On the contrary, the RPCRC cannot be recommended in this setting.

**Patient summary:**

Predicting the probability of finding prostate cancer on biopsy differed between two assessed risk calculators. After recalibration, the agreement of the models improved, and both were shown to be clinically useful.

## Take Home Message

Results from this population-based screening cohort show that the calibration of risk prediction tools need to be assessed in the local setting before using in clinical practice. The Prostate Biopsy Collaborative Group Risk Calculator provided benefit in the online version. The Rotterdam Prostate Cancer Risk Calculator needed recalibration for similar benefit.

## 1 Introduction

Accurate risk assessment is key in deciding whether or not to perform prostate biopsy in the work-up for prostate cancer (PCa). Risk prediction tools using clinical variables together with prostate-specific antigen (PSA) have been developed to improve diagnostic precision and are recommended by international guidelines [1], [2]. Two of the most used risk calculators (RCs) are the Rotterdam Prostate Cancer Risk Calculator (RPCRC), also known as the ERSPC-RC3/4, which is based on data from the Rotterdam section of the European Randomized Study of Screening for Prostate Cancer (ERSPC), and the American Prostate Biopsy Collaborative Group RC (PBCG-RC) [3], [4].

One concern with risk models is their external validity and clinical usefulness beyond the setting in which they were developed. It is common that models need to be recalibrated to fit the local setting to improve accuracy of risk predictions [5]. With increasing numbers of risk prediction tools [2], external validation, local recalibration, and head-to-head comparisons are needed to present accurate clinical recommendations.

Both the PBCG-RC and the RPCRC have been externally validated previously but never in a population-based screening cohort, and no head-to-head comparisons have been reported [2], [6], [7], [8], [9].

The introduction of magnetic resonance imaging (MRI) in PCa work-up is changing clinical practice as it has been shown to increase diagnostic accuracy [10], [11]. However, since availability of these modalities is still limited and costs might be higher than traditional work-up, risk stratification using traditional variables is still extensively used, warranting assessment of the performance of these models [12].

We aimed to test and compare the performance of the PBCG-RC and RPCRC based on discrimination, calibration, and clinical usefulness, in a screening cohort of Swedish men. Furthermore, we analyse the performance of the RCs after recalibration.

## 2 Patients and methods

### 2.1 Patient population

We used data from the Stockholm3 (STHLM3) study [13], a prospective and population-based screening study conducted in Stockholm, Sweden, between 2012 and 2015. STHLM3 enrolled 58 818 men aged 50–69 yr randomly invited by mail from the general population. Participants with PSA ≥3 ng/ml were recommended ten- to 12-core systematic prostate biopsies and prostate volume assessment measured by transrectal ultrasound (TRUS). In this study, we included all men with PSA ≥3 ng/ml who underwent prostate biopsy. Men with a prior diagnosis of PCa were excluded.

### 2.2 Risk calculation

We calculated the risk for detecting clinically significant prostate cancer (csPCa) in each participant using the RPCRC [14] and the PBCG-RC [15], both available online, and compared with biopsy outcomes. Variables used in risk predictions are shown in Table 1. One major distinction is that the RPCRC incorporates prostate volume into the risk calculation, whereas the PBCG-RC does not.

**Table 1:** Predictors incorporated in risk calculators and risk distribution for prostate cancer

	RPCRC	PBCG-RC
**Predictors**		
Age^a^	–	×
PSA^a^	×	×
DRE^a^	×	×
Prostate volume^a^	×	–
Previous biopsy^a^	×	×
Family history^a^	–	×
Race	–	×
**Distribution of risk for csPCa**		
Median, % (IQR)	3.3 (2.1–7.1)	20 (15–28)
**Distribution of risk for csPCa (recalibrated RCs)**		
Median, % (IQR)	13 (11–17)	14 (9–22)

csPCa = clinically significant prostate cancer; DRE = digital rectal examination; IQR = interquartile range; PBCG-RC = Prostate Biopsy Collaborative Group Risk Calculator; PSA = prostate-specific antigen; Race = African ancestry/other; RC = risk calculator; RPCRC = Rotterdam Prostate Cancer Risk Calculator.

a Available in our data.

Both RCs calculate the risk of detecting any PCa and csPCa (defined as Gleason score ≥7 in the PBCG-RC and Gleason score ≥7 and/or ≥T2b in the RPCRC) on systematic prostate biopsies. For our analysis, we defined csPCa as Gleason score ≥7 (International Society of Urological Pathology [ISUP] grade ≥2).

RPCRC predictions were acquired by researchers at the ERSPC, and PBCG-RC predictions were calculated using the published logistic regression model. Data on all predictors except race (African ancestry/other) were available; all participants in the STHLM3 validation cohort were classified as not of African descent.

### 2.3 Statistical analysis

We assessed discrimination (separation of those with and without csPCa), calibration (agreement between observed and predicted outcomes), and decision curve analysis (DCA; clinical benefit).

To evaluate discrimination, we plotted receiver operating curves (ROCs) for both RCs and compared the corresponding area under the curves (AUC) using DeLong’s method for two correlated ROC curves. Calibration was assessed graphically with a calibration plot and using calibration-in-the-large (intercept and slope) [16]. The intercept indicates whether predictions are systematically too high or too low, and should ideally be zero. The calibration slope reflects the average agreement between model predictions and outcomes, with a value of 1 indicating an exact match.

A DCA estimates the net benefit of a model by summing up benefits (true-positive biopsies) and subtracting harms (false-positive biopsies weighed by a factor related to the relative harm of a missed csPCa vs an unnecessary biopsy) [17]. The net benefit was evaluated for different risk thresholds for referral to biopsy, with DCA curves visualised for RC thresholds between 0 and 0.3.

Recalibration was performed using logistic regression [18] in a five-fold cross-validation loop, to avoid a positive bias due to performing recalibration and evaluation of the recalibrated model using the same dataset.

STATA 15.0 (Stata Corp., College Station, TX, USA) and R version 3.6.1 (R Foundation for Statistical Computing, Vienna, Austria) were used for data management and statistical analysis. DCA was performed using the published code [19]. Associations between clinical variables and outcomes were evaluated using Wilcoxon rank-sum tests for continuous variables and chi-square tests for categorical variables.

## 3 Results

Table 2 shows the characteristics of the study population (*n* = 5841). The median age was 64.7 yr (interquartile range [IQR] 59.8–67.6) and median PSA 4.2 ng/ml (IQR 3.4–5.7). Any PCa was diagnosed in 2248 (38%) patients and 1054 (18%) had csPCa.

**Table 2:** Population characteristics

	All	Benign biopsy	ISUP grade 1	csPCa
	(*N* = 5841)	(*N* = 3594)	(*N* = 1193)	(*N* = 1054)
**Age (yr), median (IQR)**	64.7 (59.8–67.6)	64.5 (59.7–67.6)	64.6 (59.3–67.5)	65.3 (60.8–67.7)
**PSA (ng/ml), median (IQR)**	4.2 (3.4–5.7)	4.0 (3.4–5.4)	4.1 (3.4–5.4)	5.0 (3.8–8.4)
**DRE, *N* (%)**				
**Abnormal**	571 (10)	216 (6)	101 (8)	254 (24)
**Normal**	5270 (90)	3378 (94)	1092 (92)	800 (76)
**Prostate volume (ml), median (IQR)**	42 (33–56)	45 (36–60)	40.5 (32–53)	36 (28–45)
**Previous negative prostate biopsy, *N* (%)**	458 (7.8)	372 (10)	59 (4.9)	27 (2.6)
**Family history**^a^**, *N* (%)**	810 (14)	416 (12)	212 (18)	182 (17)

csPCa = Clinically significant prostate cancer; ISUP grade ≥2; DRE = digital rectal examination; IQR = interquartile range; ISUP = International Society of Urological Pathology; PSA = prostate-specific antigen.

a First-degree relative diagnosed with prostate cancer.

### 3.1 Discrimination, calibration, and DCA

PSA alone had an AUC of 0.65 (95% confidence interval [CI] 0.63–0.67) for csPCa and was outperformed by both RCs (*p* < 0.001). The RPCRC showed slightly higher discrimination than the PBCG-RC, with AUCs of 0.74 (95% CI 0.72–0.76) and 0.70 (95% CI 0.68–0.72), respectively (*p* < 0.001).

**Fig. 1:**
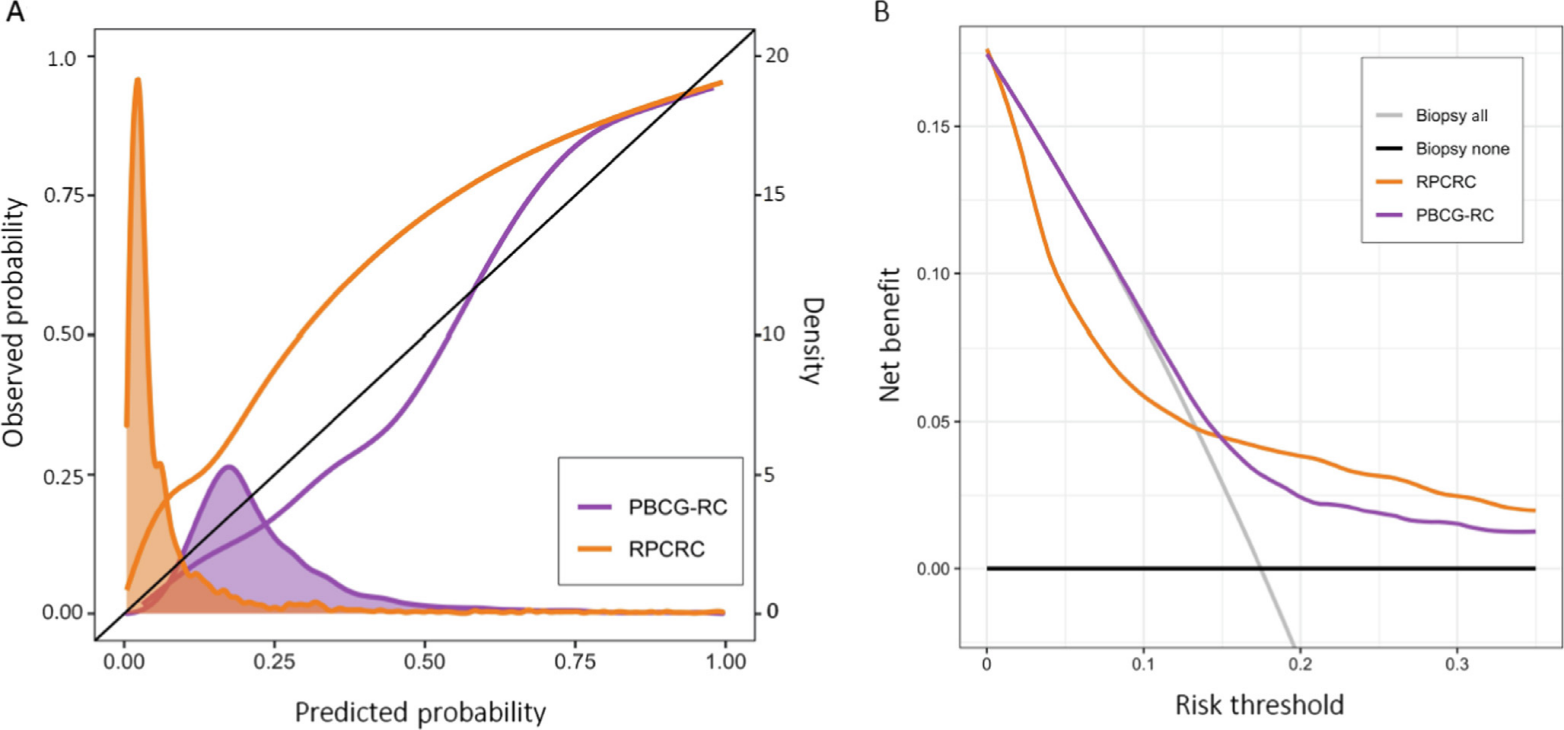
(A) Combined calibration (observed probability vs predicted probability) and density plot for risk prediction from the PBCG-RC (purple) and RPCRC (orange). The black line represents perfect calibration. The shaded areas show the distribution of predicted risk in each risk calculator. (B) Decision curve analyses demonstrating the net benefit for predicting csPCa on biopsy. Net benefits from biopsy-all (grey line) and biopsy-none (horizontal black line) strategies are shown. csPCa = clinically significant prostate cancer; PBCG-RC = Prostate Biopsy Collaborative Group Risk Calculator; RPCRC = Rotterdam Prostate Cancer Risk Calculator.

The median predicted risk of csPCa on biopsy was 3.3% (IQR 2.1–7.1%) using the RPCRC (Table 1) compared with 20% (IQR 15–28%) for the PBCG-RC, which closer matched the observed risk in the cohort (18%). The RPCRC underestimated risks up to 20%, whereas the PBCG-RC slightly overestimated the risk of csPCa at a lower observed average risk while underestimating the risk at a higher observed average risk (Fig. 1A). The calibration-in-the-large was 1.16 (95% CI 1.08–1.24) for the RPCRC and –0.37 (–0.44 to −0.30) for the PBCG-RC, and the calibration slopes were 0.73 (0.68–0.79) and 1.13 (1.03–1.23) respectively (Supplementary Table 1).

The net benefit was calculated using DCA in relation to biopsy-all and biopsy-none strategies at various risk thresholds (Fig. 1B) [17]. The PBCG-RC showed a net benefit at a threshold probability of ≥7%. The RPCRC, on the contrary, was shown to have no clinical benefit, and at risk threshold levels below 13%, it had the worse net benefit compared with the default strategy of biopsying all men with an elevated risk. The differences in the net benefit between the two RCs at risk thresholds of 5% and 10% were 0.04 and 0.02, respectively, in favour of the PBCG-RC. This translates to detecting two to four more csPCa cases per 100 biopsied men without increasing the number of unnecessary biopsies [17]. Put differently, at risk thresholds of 5% and 10%, the numbers needed to biopsy to detect an additional csPCa case were 25 and 50, respectively. At 15%, there was no difference in the net benefit between the models.

Incorporation of the measurement of prostate volume by TRUS as an invasive test adds to the harms of the RPCRC in the DCA (Supplementary Fig. 2). In the DCA, we use a test harm factor of 0.05, which is equivalent to be willing to perform 20 prostate volume measurements by TRUS to find one case of csPCa. The PBCG-RC is unaffected by this addition, as it does not include prostate volume in its risk prediction.

### 3.2 Recalibration

**Fig. 2:**
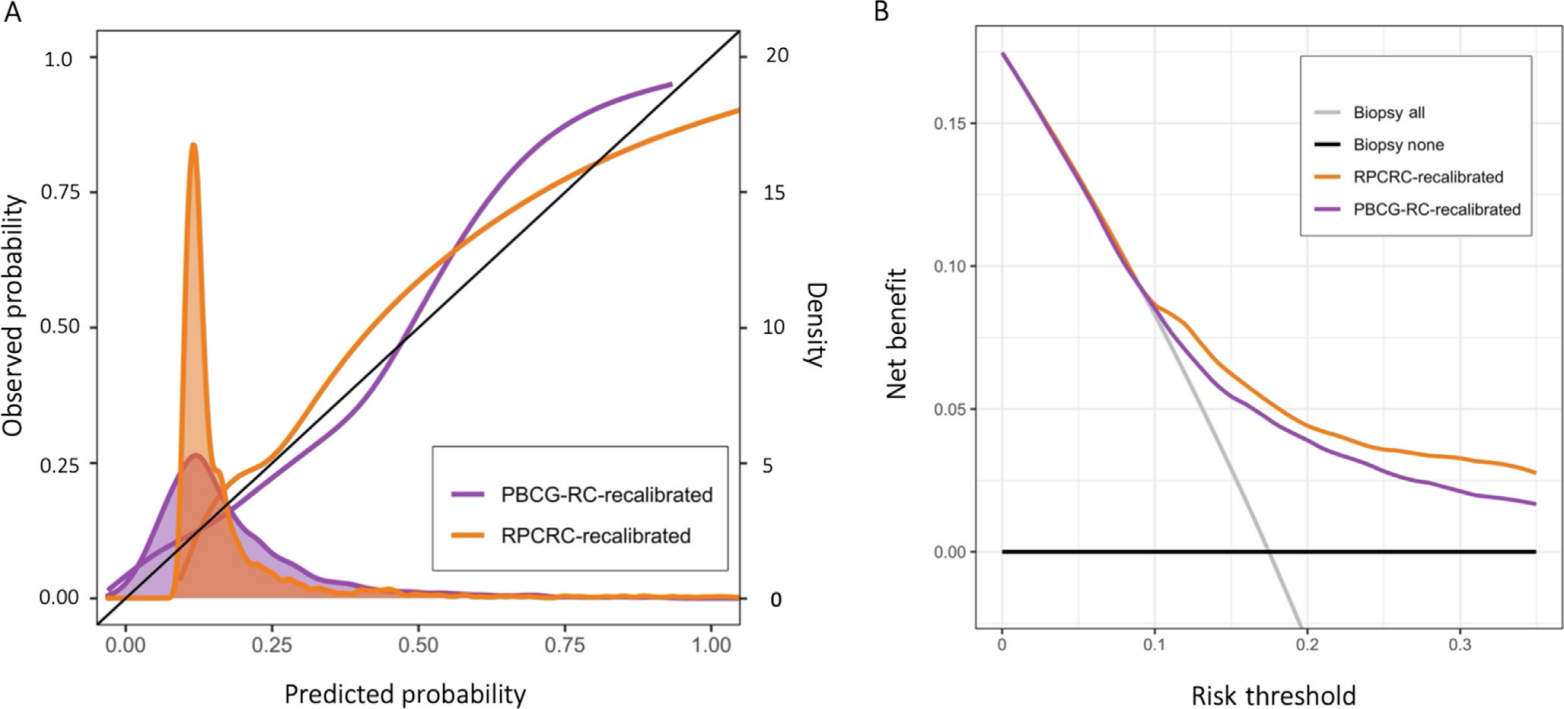
(A) Combined calibration (observed probability vs predicted probability) and density plot for the recalibrated PBCG-RC and recalibrated RPCRC. (B) Decision curve analyses demonstrating the net benefit for predicting csPCa on biopsy. Net benefits from biopsy-all (grey line) and biopsy-none (horizontal black line) strategies are shown. csPCa = clinically significant prostate cancer; PBCG-RC = Prostate Biopsy Collaborative Group Risk Calculator; RPCRC = Rotterdam Prostate Cancer Risk Calculator.

Recalibration by adjusting the intercept shifted the median risks in the RPCRC to 13% (IQR 11–18%) and in the PBCG-RC to 14% (IQR 9–22%), without affecting the risk distribution (Table 1). Recalibration enhanced the performance of both RCs, with a closer correlation between predicted and observed probabilities (Fig. 2A and Supplementary Table 1), and improved the net benefit (Fig. 2B).

### 3.3 Clinical effects

We assessed clinical effects by calculating the numbers of avoided nonsignificant PCa (ISUP grade 1) and missed csPCa at fixed levels (25%, 33%, and 50%) of avoided biopsies for the original and recalibrated RCs (Table 3). Our results show that 25% of biopsies could be saved, avoiding 21% (RPCRC) and 19% (PBCG-RC) nonsignificant PCa cases, at the expense of missing 9% and 10% csPCa cases, respectively. For the recalibrated RCs, 21% (RPCRC) and 24% (PBCG-RC) nonsignificant PCa cases would be avoided, and 10% and 11% csPCa cases, respectively, were undetected at the level of 25% fewer biopsies performed.

**Table 3:** Clinical effects of using the RPCRC and the PBCG-RC standardised per 1000 biopsied men

	Avoided biopsies (%)	Biopsied men, *n*	Avoided ISUP grade 1, *n* (%)	Missed csPCa, *n* (%)	Risk threshold (%)^a^
Biopsy all men with PSA ≥3 ng/ml	0	1000	0/204 (0)	0/180 (0)	–
RPCRC	25	754	43 (21)	15 (9)	2.1
	33	685	61 (30)	22 (12)	2.4
	50	494	99 (48)	46 (25)	3.3
PBCG-RC	25	784	39 (19)	18 (10)	15
	33	685	61 (30)	30 (17)	17
	50	520	94 (46)	50 (28)	20
Recalibrated RPCRC	25	738	45 (22)	18 (10)	11
	33	677	61 (30)	24 (13)	12
	50	507	96 (47)	45 (25)	13
Recalibrated PBCG-RC	25	766	43 (21)	20 (11)	9
	33	663	65 (32)	33 (18)	11
	50	510	96 (47)	52 (29)	14

csPCa = clinically significant prostate cancer, ISUP grade ≥2; ISUP = International Society of Urological Pathology; PBCG-RC = Prostate Biopsy Collaborative Group Risk Calculator; PSA = prostate-specific antigen; RPCRC = Rotterdam Prostate Cancer Risk Calculator.

a Risk threshold of finding clinically significant prostate cancer on biopsy, that is, cut-off risk score in the RPCRC and the PBCG-RC for biopsy recommendation.

Setting the risk cut-off for biopsy recommendation at 4%, as proposed by the ERSPC [14], would result in avoiding 57% of biopsies and missing 30% of csPCa cases in the original RPCRC. The PBCG-RC does not suggest a specific cut-off value but rather an individualised approach based on shared decision-making. However, using the same 4% cut-off for the PBCG-RC, almost all men (5837 out of 5841) would be referred for biopsy. At a risk cut-off of 15%, the PBCG-RC would save 25% of biopsies and leave 10% csPCa cases undetected.

Owing to the narrow risk distribution in the RPCRC (Fig. 1A), small shifts in the risk threshold led to marked clinical effects. The difference in risk cut-off for biopsy to avoid 25% or 50% of biopsies is only 1.2% (2.1% vs 3.3%) and results in missing 17% more men with csPCa (Table 3). Maintaining a 4% risk cut-off for biopsy referral, all men would be recommended biopsy using the recalibrated RPCRC and 95% using the recalibrated PBCG-RC.

### 3.4 Agreement between RCs

Finally, we compared the calculated risk for each individual between RCs. Spearman’s correlation coefficient (*r*) was 0.55, and there was significant variance (Supplementary Fig. 1). Risk estimates differed by up to a factor of 10 between RCs, and in only 2117 (36%) out of 5841 men, the RCs would be concordant on recommendation for biopsy or not (cut-off ≥4%). Setting the cut-off at 10%, the RCs showed even greater disagreement: 23% of the participants would then receive the same recommendation. After recalibration, the RCs agreed in 92% of the cases using a 4% risk threshold and in 75% using a 10% risk threshold.

## 4 Discussion

RCs ameliorate selection of men for prostate biopsy but have been shown to lack external validity in part due to differences in population characteristics, biopsy technique, and selected set of predictors in RCs [9], [20]. This study is the first external validation of these popular RCs in a large population-based screening cohort. Our results show that both RCs have good discriminatory abilities, but the RPCRC needed recalibration to be clinically useful. Prior to recalibration, the RPCRC underestimates risk and would result in missing a considerable number of csPCa cases. We also find that a significant proportion of men would get contradictory biopsy recommendations from these two RCs.

A notable difference between the RCs is that prostate volume is not incorporated in the current version of the PBCG-RC. PSA density calculated by PSA and prostate volume has been shown to be a strong predictor of PCa [21], and the higher discrimination in the RPCRC is likely attributed to the inclusion of volume in its risk calculation. However, additional work-up (TRUS or MRI) is required to obtain information on prostate volume, and models requiring prostate volume therefore might have lower usability for initial management of patients. Indeed, incorporating measurement of prostate volume as cost or harm [17] worsened clinical benefit of both the original and the recalibrated RPCRC in the DCA (Supplementary Fig. 2).

### 4.1 ERSPC cohort

Similar to the STHLM3 study, the Rotterdam section of the ERSPC study constitutes a screening population, where men were invited and followed over time with regular PSA testing, and biopsied if PSA reached ≥3 ng/ml or there were abnormal findings on digital rectal examination [22]. However, there were several differences between these cohorts (population characteristics of developing cohorts are shown in Supplementary Table 2). The proportion of participants undergoing biopsy was 23% in the ERSPC versus 11% in the STHLM3 study, and cancer detection rates differed. In the STHLM3 study, 18% had ISUP grade ≥2 on biopsy compared with 9% for biopsy-naïve men and 5% for men with a prior negative biopsy in the ERSPC trial. This is probably partly due to the larger proportion of biopsied men and the outdated sextant biopsy technique being employed in ERSPC, in contrast to the ten-to 12-core standard used in STHLM3. Detection rates of csPCa have been shown to be considerably higher in men undergoing 12 biopsies than in those undergoing six biopsies [6].

Another factor affecting the proportion of csPCa cases is that the ERSPC biopsies were all denoted a Gleason score prior to the 2005 updated ISUP guidelines [23]. Hence, some biopsies, which are classified as Gleason 7 today, might have been classified as Gleason 6 in the ERSPC study.

Further, age, an established risk factor for PCa, is not used as a predictor in the RPCRC. However, the age distribution in our study was similar to the Rotterdam section of the ERSPC study (median 64.7 and 64.1 yr, respectively). Therefore, discrepancies in risk assessment attributed to age are probably minor.

### 4.2 PBCG cohort

The PBCG-RC is based on biopsies from 5992 men visiting North American centres for PCa testing and follow-up [3]. Although there was no difference in age (median 64.7), the PBCG cohort had notably higher median PSA (6.0 vs 4.2 ng/ml), higher proportions of abnormal DRE (28%), family history (18%), and prior negative biopsy (22%; Supplementary Table 2). Positive biopsies were found in 50% of men and csPCa in 32%. This indicates that the distribution of underlying PCa risk was higher in the PBCG than in the STHLM3 and ERSPC studies, and illustrates the marked differences between clinical cohorts (patients referred to urologists for prostate biopsy) and screening cohorts.

### 4.3 Clinical usefulness

In concordance with previous studies, we show that the use of RCs can improve the diagnostic accuracy for predicting prostate biopsy outcome as compared with a PSA-only strategy [2], [3], [4], [5], [6], [7], [8], [24]. From a clinical perspective, risk predictions are most useful at lower risk thresholds, as high-risk patients would probably be recommended biopsy without the aid of a risk prediction tool. Therefore, the poor calibration of the PBCG-RC at a ≥50% risk threshold is less of a problem than that of the RPCRC at 10% in a clinical setting.

Reducing the number of unnecessary biopsies is imperative to minimise associated adverse events, as well as decreasing overdetection of nonsignificant PCa. However, with fewer biopsies performed, some clinically significant cancers will inevitably remain undetected. Thus, increasing the risk threshold for recommending biopsy will reduce the number of biopsies at the cost of missed cancer.

We believe that there might be no ideal risk cut-off but rather that each case must be discussed individually, and the shared decision-making between patient and physician will be influenced by many factors, including the risk averseness of the patient as well as the predictive accuracy of a diagnostic test.

Previous evidence shows that the risk of finding csPCa on biopsy in men with PSA ≥3 ng/ml is approximately 10% [13]. Since PSA 3–4 ng/ml is widely accepted as a cut-off threshold for biopsy, one could argue that a 10–15% risk threshold is a more clinically relevant cut-off than the 4% recommended by ERSPC. At 15% risk cut-off, the online version of the PBCG-RC showed clinical effects similar to other risk prediction tools and biomarkers, avoiding 25% of biopsies at the expense of leaving 10% csPCa undetected [13], [25], [26]. The RPCRC showed similar clinical benefits at 11% risk cut-off after recalibration (Table 3).

The substantial differences in assessed risk between RCs presented in this study are mainly explained by poor calibration, as illustrated by the high agreement between recalibrated RCs, and risk distribution. Most men using the uncalibrated RPCRC would not reach the risk threshold for biopsy, whereas many of them would undergo biopsy using the PBCG-RC. These results demonstrate how decision-making, on an individual basis, will be dependent on which RC is used. Almost two-thirds of men in our study would get contradictory recommendations whether to biopsy or not using uncalibrated RCs. With calibration and narrow risk distribution being the main problems, our data show that the current online version of the RPCRC cannot accurately predict PCa risk in this screening cohort and therefore should not be recommended in clinical decision-making without prior recalibration.

Our study is not without limitations. First, despite the rapid shift to MRI-guided techniques, a systematic prostate biopsy protocol was used in this study. However, MRI is not readily available in all settings, and there might be cost-related issues. In addition, these RCs are widely used in everyday clinical practice, available online, and recommended in guidelines. Therefore, we found it important to test their accuracy. Second, we have missing data on race, which is one of the predictors used in the PBCG-RC. We base the assumption that no participants had African ancestry on current demographics (<0.1% African or American born men aged 50–69 yr in the Stockholm region during 2012–2015) [27]. Finally, extrapolation of our findings to other populations should be made with caution; rather, this study serves to illustrate the importance of regional calibration of risk prediction tools to the target population, although we acknowledge that it may not be feasible to recalibrate for every single subpopulation or clinic. Our study does not solve the problem with generalisability and calibration of risk prediction tools, and making recalibration a clinically applicable method remains a challenge.

## 5 Conclusions

In conclusion, the results from this large population-based screening cohort demonstrate significant intraindividual disagreement between the assessed RCs. The PBCG-RC provided clinical benefit in the version available online, whereas the RPCRC showed no net benefit in this setting at recommended threshold levels and should not be used in clinical practice if not recalibrated. This study illustrates that for risk prediction tools to be clinically reliable and safe, assessment of calibration is essential.

  ***Author contributions*:** Jan Chandra Engel had full access to all the data in the study and takes responsibility for the integrity of the data and the accuracy of the data analysis.

*Study concept and design*: Chandra Engel, Nordström, Eklund, Grönberg.

*Acquisition of data*: Chandra Engel, Nordström, Remmers, Chellappa, Egevad.

*Analysis and interpretation of data*: Chandra Engel, Nordström, Eklund, Palsdottir, Remmers, Ankerst.

*Drafting of the manuscript*: Chandra Engel, Nordström, Eklund.

*Critical revision of the manuscript for important intellectual content*: Chandra Engel, Nordström, Eklund, Palsdottir, Mortezavi, Remmers, Ankerst, Grönberg.

*Statistical analysis*: Chandra Engel, Nordström, Eklund, Palsdottir.

*Obtaining funding*: Nordström, Eklund, Grönberg.

*Administrative, technical, or material support*: Nordström, Eklund, Grönberg.

*Supervision*: Nordström, Grönberg, Eklund.

*Other*: None.

  ***Financial disclosures:*** Jan Chandra Engel certifies that all conflicts of interest, including specific financial interests and relationships and affiliations relevant to the subject matter or materials discussed in the manuscript (eg, employment/affiliation, grants or funding, consultancies, honoraria, stock ownership or options, expert testimony, royalties, or patents filed, received, or pending), are the following: None.

  ***Funding/Support and role of the sponsor*:** Grants were received from Swedish Research Council (Vetenskapliga Rådet), Region Stockholm, and the Swedish Cancer Society (Cancerfonden).
