# Supplementary material for: External Validation of the Prostate Biopsy Collaborative Group Risk Calculator and the Rotterdam Prostate Cancer Risk Calculator in a Swedish Population-based Screening Cohort
Source: Eur Urol Open Sci. 2022 May 19;41:1–7. doi: 10.1016/j.euros.2022.04.010 (PMC9257644; doi:10.1016/j.euros.2022.04.010)
Supplement: Supplementary data 1 [file mmc1.docx]

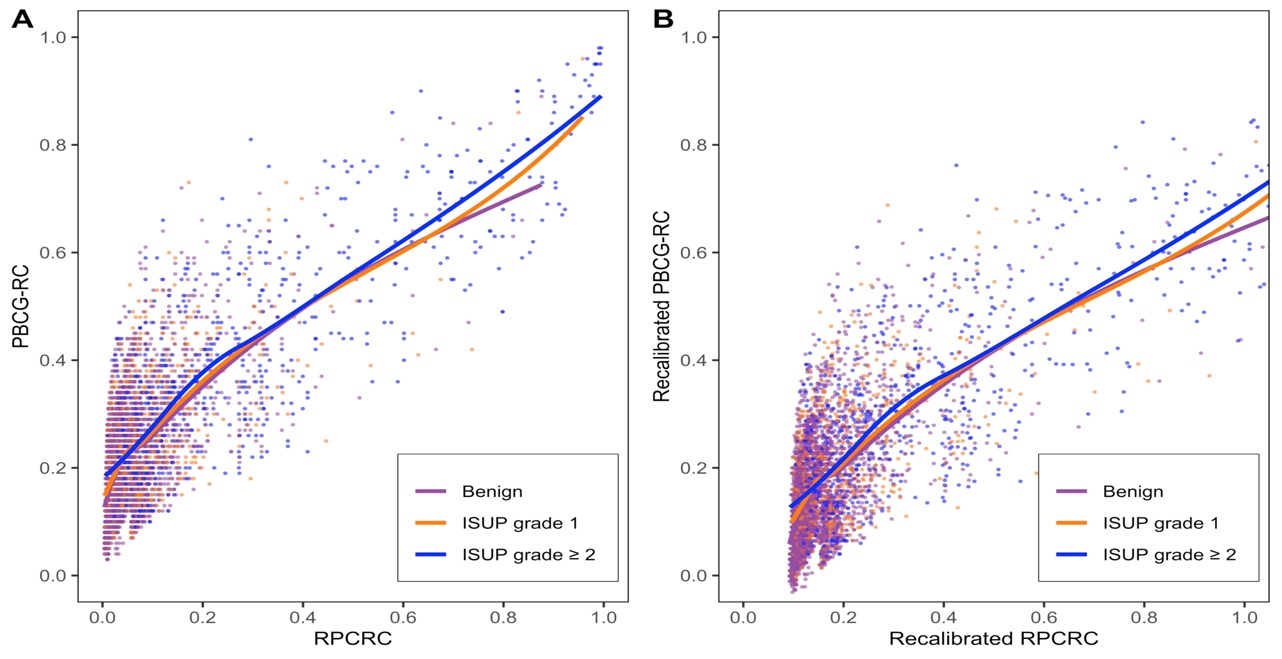


**Supplementary Figure 1**. A: Correlation between predicted risk in each individual participant calculated by the PBCG and the RPCRC, Spearman’s r = 0.55, p-value < 0.001, p-value < 0.001. B: Correlation between recalibrated PBCG and recalibrated RPCRC, Spearman’s r = 0.55, p-value < 0.001. The lines represent the smoothing function. ISUP: International Society of Urological Pathology. PBCG-RC: Prostate Biopsy Collaborative Group Risk Calculator. RPCRC: Rotterdam Prostate Cancer Risk Calculator.

| Risk Calculator | Observed Proportion | Predicted Proportion* | Calibration Slope (95% CI) | Intercept Value (95% CI) | AUC (95% CI) |
| --- | --- | --- | --- | --- | --- |
| PBCG-RC | 0.18 | 0.24 | 1.13 (1.03-1.23) | -0.37 (-0.44-(-)0.30) | 0.70 (0.68-0.72) |
| RPCRC |  | 0.08 | 0.73 (0.68-0.79) | 1.16 (1.08-1.24) | 0.74 (0.72-0.76) |
| Recalibrated PBCG-RC |  | 0.18 | 0.93 (0.85-1.02) | 0.00 (-0.07-0.07) | - |
| Recalibrated RPCRC |  | 0.17 | 1.17 (1.07-1.28) | 0.01 (-0.06-0.09) | - |

**Supplementary Table 1**. Numerical evaluation of the calibration of the risk calculators. Ideal calibration would have intercept 0, and slope 1. Observed proportion corresponds to 18% clinically significant prostate cancer on biopsy in the study population. AUC: Area under the curve. * Mean of the predictions.

|  | ERSPC RC3 | ERSPC RC4 | PBCG-RC | STHLM3 |
| --- | --- | --- | --- | --- |
|  | N=3616 | N=2215 | N=5992 | N=5841 |
| Age, years (median, IQR) | 66 (61-70) | 67 (63-71) | 64.7 (59.0-69.0) | 64.7 (59.8-67.6) |
| PSA, ng/ml (median, IQR) | 4.3 (3.1-6.4) | 4.5 (3.5-6.1) | 6 (4.4-9.0) | 4.2 (3.4-5.7) |
| DRE (N, %) |  |  |  |  |
| Abnormal | 1279 (35) | 478 (22) | 1698 (28) | 571 (10) |
| Normal | 2337 (65) | 1737 (78) | 3416 (57) | 5270 (90) |
| Unknown | 0 (0) | 0 (0) | 878 (15) | 0 (0) |
| Prostate Volume, ml (median, IQR) | 41 (32-55) | 48.3 (37.5-61.7) | Not available | 42 (33-56) |
| Previous negative prostate biopsy (N, %) | 0 (0) | 2215 (100) | 1346 (22) | 458 (8) |
| Family history* (N, %) | Not available | Not available | 1095 (18) | 810 (14) |

**Supplementary Table 2**. Population characteristics in the development cohorts (PBCG and first and second screening rounds in the Rotterdam section of the ERSPC) of the RPCRC (ERSPC RC3/4) and PBCG-RC and the validation cohort (all biopsied men with PSA >3 ng/ml in STHLM3). DRE: Digital rectal examination. ERSPC RC: European Randomized Study of Screening for Prostate Cancer Risk Calculator. PBCG-RC: Prostate Biopsy Collaborative Group Risk Calculator. RPCRC: Rotterdam Prostate Cancer Risk Calculator. STHLM3: Stockholm-3 study cohort. *First degree relative diagnosed with prostate cancer.


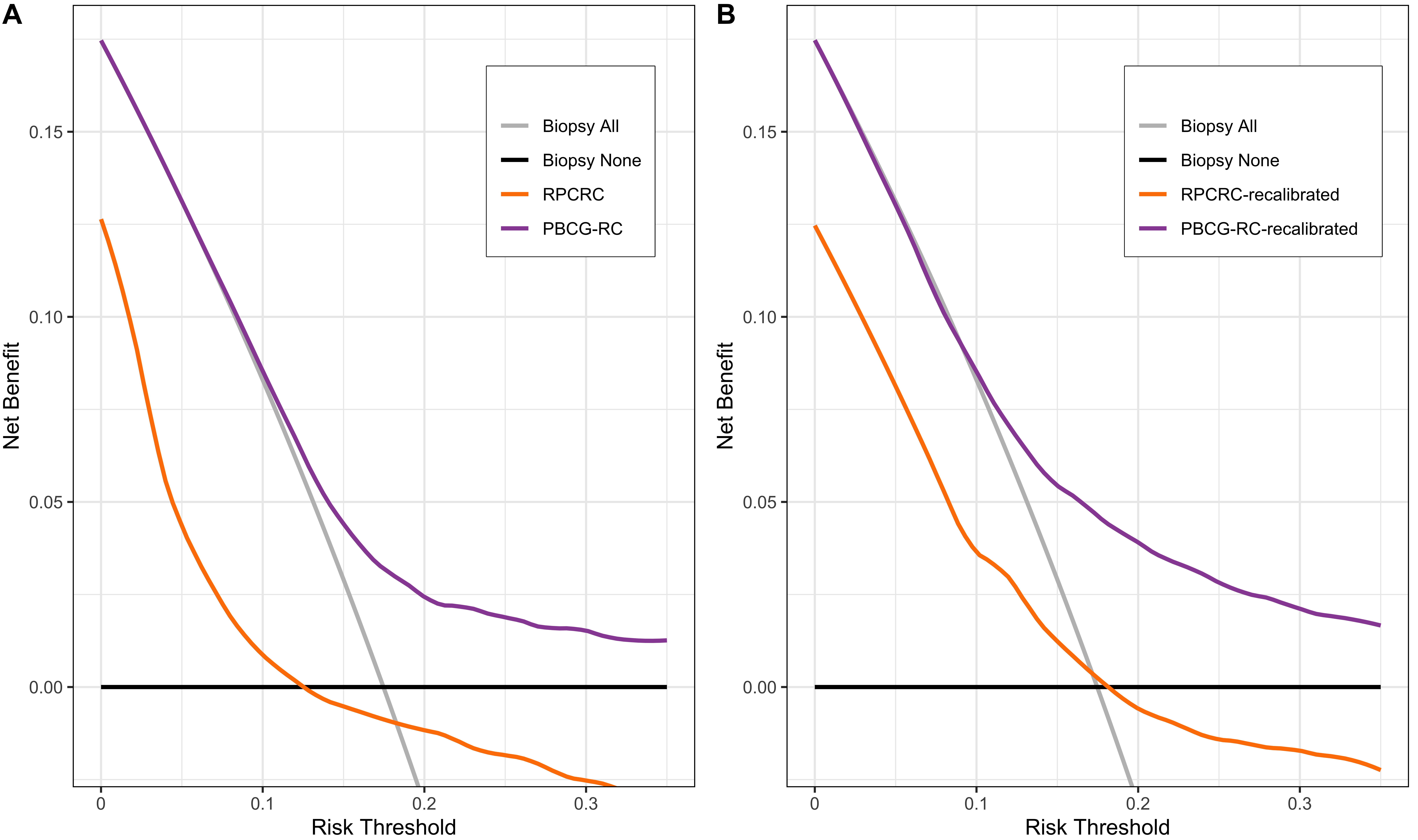


**Supplementary Figure 2**. Decision curve analyses incorporating prostate volume measurement as test procedure harm. Test harm factor was estimated to be 0.05 which translates to the acceptance of performing up to 20 transrectal ultrasound prostate volume measurements to find one clinically significant prostate cancer. (A) Decision curve demonstrating the net benefit for predicting clinically significant prostate cancer on biopsy for original risk calculators and (B) for recalibrated risk calculators. Net benefits from biopsy-all (grey line) and biopsy-none (horizontal black line) strategies are shown. PBCG-RC: Prostate Biopsy Collaborative Group Risk Calculator. RPCRC: Rotterdam Prostate Cancer Risk Calculator.
